# Digestive Organs

**Published:** 1906-02-17

**Authors:** 


### DIGESTIVE ORGANS.

**Cancer of the Stomach.**—Cancer of the stomach is sometimes easy, but frequently is difficult, to diagnose, and in view of the importance of early diagnosis it is not surprising that many methods have been suggested by clinical pathologists to aid or reinforce doubtful physical signs and symptoms. McIntosh [1] has summarised some of these, which depend for the most part on an analysis of the stomach contents or an examination of the fæces for minute traces of blood. The latter method has already been described in these columns, and although, as McIntosh points out, it is true that traces of blood in the fæces are practically constant in gastric carcinoma, the value of the test is much diminished by the fact that they are found, at least intermittently, in many other conditions. The absence of blood, however, on repeated examination may perhaps fairly be taken as negativing cancer of the stomach wall. Cancer may, however, be present in adjacent organs, and if a tumour is felt McIntosh suggests that inflating the stomach may assist in locating the exact attachments of the lump. The examination of the gastric contents is often of value. McIntosh, out of four suggested methods, is inclined to regard two as reliable. One is Salomon's albumen test, which consists in carefully washing out the stomach with salt solution and testing for albumen and nitrogen. The patient is prepared by 24 hours on liquid diet, and the last meal taken eight hours before the stomach washing must contain no nitrogenous material. The presence of albumen is said to indicate cancer. The other test is Gluzinki's. This depends upon a chronic mucous gastritis developing in cases of cancer. When this occurs, free HCl is found after food, but not fasting, and a long bacillus is also often present. This bacillus is destroyed by strong HCl, but can live in presence of lactic acid. The diagnostic value of its presence is therefore the same as that of lactic acid. McIntosh holds that the absence of free HCl and presence of lactic acid, especially when albuminous food is badly digested, are practically indicative of cancer, but insists on the importance of repeated examinations.

**Gastric Ulcer.** — For some time it has been suspected that many widely dissimilar conditions are grouped together under this heading. Dawson [2]

distinguishes two types—the acute perforating ulcer and the chronic indurated ulcer. The latter is the common kind found in anæmic young women, while the former is common to either sex, may occur at any age, and is often rapidly fatal. But not all cases presenting the features of chronic ulcer are really due to this cause. Epigastric pain related to food, vomiting, and localised tenderness, even when associated with considerable hæmatemesis, may be due to at least four other causes: (1) anæmic dyspepsia in young women; (2) septic gastritis from oral or pharyngeal sepsis; (3) hyperchlorhydria; (4) neurasthenic dyspepsia. The diagnosis of these conditions from ulcer can only be made by a careful analysis of the symptoms. The important points are (1) the protracted cases in anæmic girls where the hæmatemesis and other symptoms rapidly yield to rest in bed and dieting are usually not ulcer. Very profuse hæmorrhage, especially when the other symptoms have subsided, is in favour of ulcer. The pain is not usually relieved by food in ulcer except when it is near the pylorus; in hyperchlorhydria and neurasthenia, the pain is often relieved by food. Deep very localised tenderness, especially when persisting after other symptoms have disappeared, is in favour of ulcer. It has, however, no relation to its actual position. Superficial (skin) tenderness is not always present in ulcer, and occurs in other conditions. If persisting after the other symptoms, and if at the same time its area contracts, it is more likely to be due to ulcer. Increase of HCl is much in favour of ulcer, but is also found in neurasthenia. In anæmic dyspepsia it is not increased, and in septic gastritis it is diminished. If on examination of the gastric contents free nuclei of epithelial cells and leucocytes are found free HCl is present; if, on the other hand, these cells are intact, it is absent. The large non-motile bacilli (above described) go with the presence of lactic acid and stagnation of stomach contents, and in presence of HCl point to benign stenosis of the pylorus. If there are no signs of HCl cancer is probable. Pus and blood-cells point to ulceration. Here again cancer may be diagnosed by the absence of HCl.

**Acid Dyspepsia.**—This is considered by Hutchison [3] the commonest form of dyspepsia among men, and is divided by him into three stages. Firstly, one of intermittent hyperchlorhydria, and characterised by attacks of pain, usually some hours after food, and frequently relieved by taking more. The pain is due to over-sensitiveness, as well as hypersecretion. The second stage is one of chronic continuous dyspepsia, in which the fasting stomach contains acid gastric juice; in this stage pyloric spasm is developed, in which severe cramp-like pain in the epigastrium or to the right of it comes on some time after food, and continues for some hours. The third stage is that of dilatation of the stomach, possibly complicated by ulcer or benign stenosis of the pylorus. In this stage there will be the physical signs of dilatation, often accompanied by constipation, enlargement of the liver, and emaciation. The treatment consists of alkalies, preferably magnesium carbonate in the first stage, rest in bed, hot poultices, milk diet, and alkalies in the second stage; and then a continuation of treatment as in the first; while in the third stage gastric lavage, and possibly gastro-enterostomy, will be necessary.

**Hydrochloric Acid.**—It will be noted what an important part the presence or absence of free HCl plays in the diagnosis of the conditions enumerated above. The idea naturally suggests itself that an improvement in symptoms at any rate might be obtained by the administration of acids in conditions when its secretion is diminished. Acids cannot, however, be given in sufficient quantities to make up for the deficiency, and it is thought that their value, which seems commonly accepted, may be due to some stimulant or antiseptic action. The latter view may be supported by the fact alluded to above that the large bacilli accompanying lactic acid are destroyed by HCl. Chase [4] has observed the effect of giving this acid in three cases. To the first, a woman with a dilated stomach and gastric catarrh, 15 minims of a 10 per cent. solution of HCl were given twice at intervals of 15 minutes after food. The symptoms improved, but the already small amount of HCl secreted was diminished. The second case was apparently one of nervous dyspepsia, with deficiency of HCl; there was no dilatation. In this case no improvement followed, though the total acidity was always increased, and free HCl was sometimes found. In the third case, similar to the first in symptoms, 20 minims after each meal effected a cure. It is difficult to draw any conclusions from so few cases, but Chase's results seem in general agreement with the usual view that acids do good in cases of "nervous" dyspepsia, even when some organic change has taken place in the stomach walls.

[1] Med. Rec., Oct. 21, 1905, p. 655. [2] Brit. Med. Jour., Oct. 21, 1905, p. 1032. [3] Clin. Jour., Aug. 16, 1905, p. 273. [4] Brit. Med. Jour., Oct. 7, 1905, p. 893.

*(To be continued.)*

